# Diagnostic Accuracy of Point-of-Care Tests Measuring Glycosylated Haemoglobin (HbA1c) for Glycemic Control: A Field Study in India

**DOI:** 10.7759/cureus.17920

**Published:** 2021-09-13

**Authors:** Sagar Khadanga, Gyanendra Singh, Abhijit P Pakhare, Rajnish Joshi

**Affiliations:** 1 Internal Medicine, All India Institute of Medical Sciences, Bhopal, Bhopal, IND; 2 Community and Family Medicine, All India Institute of Medical Sciences, Bhopal, Bhopal, IND

**Keywords:** hba1c, diagnostic accuracy, point of care, diabetes, glycaemic control

## Abstract

Objectives

This study was performed to estimate diagnostic accuracy of the two commercially available point-of-care tests to identify poor glycemic control defined by HbA1c levels, with high-performance liquid chromatography (HPLC) as a reference.

Settings

The study was carried at two locations, general medical outpatient department of a teaching medical college in Bhopal (urban), and a primary health care centre in a rural area in the state of Madhya Pradesh, India.

Participants

All individuals with diabetes mellitus who presented to the health care facility for assessment of glycemic control. We compared HbA1c estimated from two index tests (Hemocue Hb 501, Sweden; SD Biosensor, South Korea) from capillary blood samples with HPLC performed from venous blood, as a reference standard.

Primary and secondary outcome measures

Diagnostic properties of index tests such as sensitivity, specificity, positive and negative predictive value and diagnostic accuracy for identifying poor glycemic control were primary outcome measures. Lin’s concordance correlation coefficient (CCC) was secondary outcome measure.

Results

Out of 114 patients, all received reference standard - 103 patients received Hemocue A1C test and 110 patients received SD Biosensor test. Overall both the index tests had similar diagnostic accuracy estimates. The area under the Receiver Operating Curve for SDA1c device was 0.935 (95% CI 0.886-0.983), and for Hemocue device was 0.938 (95% CI 0.893-0.984). The Hemocue device HbA1c value of above 7.0 (positive) correctly predicted poor glycemic control 92% times (81.58% for SD device). There were 4 vs. 11 device failures and 14 vs. 12 failures with SD and Hemocue, respectively. Ambient air temperatures were no different for the device test failures.

Conclusions

Commercially available point-of-care tests evaluated in this study are comparable and an acceptable alternative to HPLC-based measurements for the assessment of glycemic control. Tests and device failure rates of both the index tests are similar.

## Introduction

Diabetes is a worldwide health concern characterized by elevated blood glucose levels caused by inadequate pancreatic insulin synthesis and/or decreased insulin sensitivity [1]. The prevalence of diabetes rapidly rising in India increased from 26.0 million in 1990 to 65.0 million in 2016 [2]. The total number of people with diabetes is projected to rise from 31.7 million in 2000 to 79.4 million in 2030 [3]. Early detection of diabetes and its consequences allows for prompt and effective preventive therapy [4], which helps to prevent the development or progression of macrovascular and microvascular illness. The key to avoiding diabetes problems is to keep blood glucose levels within normal limits in addition to lifestyle modifications, blood pressure control, and dyslipidemia management, etc. [5]. The reference measure of glycaemic management in people with diabetes is determining the glycated haemoglobin A1c (HbA1c) levels. It reflects the glycemic level throughout the previous two to three months [6]. The use of HbA1c in the management of diabetes has been well established since the Diabetes Control and Complications Trial (DCCT) and the UK Prospective Diabetes Study (UKPDS) trial [7-8]. American Diabetes Association (ADA) in 2010 updated their guidelines for the diagnosis of diabetes, taking HbA1c into account. Since then, cut-offs of 6.5 percent have been utilised as a handy marker for both diagnosis and follow-up of diabetic patients [9-10].

Various methods are available for the measurement of HbA1c levels, separate HbA1c from other types of hemoglobin using charge differences (e.g., ion-exchange high-performance liquid chromatography [HPLC], electrophoresis or isoelectric focusing), structural difference (e.g., affinity chromatography or immunoassay), or chemical analysis (e.g., photometry and spectrophotometry) [11-14]. More than 30 different methods have been described to estimate HbA1c. Due to the inconsistency in the technique used and reporting format, ADA recommends the International Federation for Clinical Chemistry (IFCC) units (mmol/mol) and derived the National Glycohemoglobin Standardization Program (NGSP) units (%) using the IFCC-NGSP master equation. The methods certified by the NGSP, a US-based organization, relate individual techniques to HbA1c values as obtained in the landmark DCCT trial [15]. The most commonly used techniques are ion-exchange high-performance liquid chromatography (HPLC), capillary chromatography, borate affinity assay, and immunoassay; HPLC is considered the gold standard [11]. However, it has greater technical needs, including equipment, expenditure, and longer turnaround times. In the past few years, the concept of point-of-care (POC) HbA1c testing has emerged [16]. Commonly used POC, HbA1c assays use the principles of borate affinity or immunoassay. In borate affinity assays glycated hemoglobin binds to borate resins, with the potential to overestimate HbA1c, as the technique is not HbA1c specific [11]. In contrast, immunoassay uses anti-HbA1c antibodies against the glycated N-terminal of a beta-hemoglobin chain. While this technique is attractive, interference with other hemoglobin chains remains a concern.

Previous HbA1c diagnostic accuracy studies were conducted in high-income countries that have more controlled environmental and logistic conditions for the storage of test kits and operation of the equipment. Ours is the first study conducted in public healthcare settings of a tropical middle-income country. The current study was designed to answer two research questions: a) Among individuals with diabetes mellitus, point-of-care (POC) HbA1c measurement devices (based on immunoassay or borate affinity principle), as compared to HPLC as a reference standard, are accurate for assessing optimal glycemic control (HbA1c ≤ 7%); b) In a community-based setting in a rural area, the use of a POC device to estimate the HbA1c level is feasible. We aimed to compare the accuracy and feasibility of two commercially available point-of-care devices. This article was previously posted to the *Preprints* preprint server on January 29, 2019.

## Materials and methods

Design and setting

This was a cross-sectional diagnostic accuracy and feasibility study. The study was conducted at two locations, the medical outpatient department at the All India Institute of Medical Sciences Bhopal, Madhya Pradesh (Urban), and Centre for Rural Health AIIMS (CRHA) located at the Primary Health Centre at Chiklod, Madhya Pradesh (Rural). Individuals with diabetes mellitus who sought care at both facilities were evaluated for glycemic control.

Participants

The study sought to include all individuals with diabetes mellitus who presented to the health care facility for the assessment of glycemic control. The individuals were previously diagnosed with diabetes (based on ADA criteria: fasting plasma glucose of 126 mg/dL or above or post-meal plasma glucose of 200 mg/dL or above or HbA1c level of 6.5% or above) at least three months before the date of inclusion in the study. Written informed consent was obtained from all the eligible participants. For logistic reasons, the participants were sampled from these facilities once weekly for the study duration.

Ethics issues and permissions

The study design was approved by the Institutional Human Ethics Committee of All India Institute of Medical Sciences, Bhopal (Ref: IHEC-LOP/2015/IM0056). Participant Information Sheet in Hindi language was provided to each participant. All participants provided written informed consent prior to initiation of any study procedures. Participants were enrolled after obtaining written informed consent. The results of biochemical investigations were communicated to the participants and were appropriately managed or referred.

Study procedures

All eligible and consenting participants were administered a questionnaire to collect information about demographics, duration of diagnosis of diabetes mellitus, current therapies, and any history of hemoglobinopathy. Subsequently, the following samples for the index test and reference standard were collected within a 10-minute interval of each other. We first collected a 2-mL venous sample in an ethylenediaminetetraacetic acid (EDTA) tube, which was immediately stored between 4 and 80°C in an ice-pack-containing vaccine carrier. These samples were transported to the laboratory on the same day in a temperature-controlled environment for HPLC-based HbA1c estimation (Reference Standard). The test was performed in a National Accreditation Board for Testing Calibration Laboratories (NABL) accredited laboratory. Then we collected capillary blood samples using a finger-prick. We collected one drop of capillary blood (approximately 5 μL) for each of the index tests: a Borate affinity-based point-of-care test (Hemocue A1c 501, Sweden) and an immunoassay-based point-of-care test (SD A1c care Bioassay, South Korea). The characteristics of the two index tests are presented in Table 1. Point-of-care tests were performed in front of the patient, using standard techniques as per the manufacturer’s guidelines. Briefly, in the borate affinity meter (Hemocue A1c 510) a blood sample was collected using a reagent pack (which draws appropriate volume into the reagent pack), and the reagent pack is inserted into the cartridge. The cartridge was then inserted into the machine, and the results are displayed on the meter in three minutes. In the immunoassay meter (SD A1c care) a drop of blood (about 5 μL) is collected in a reagent tube, and allowed to mix for a minute. A test strip is inserted into the meter, and 5 μL of the blood-reagent mixture is applied to the sample port on the strip. The results were obtained in three minutes. Both the index tests were conducted, and interpreted, blinded to the results of the reference standard. The HPLC results were available only the next day.

**Table 1:** Characteristics of the two index tests IFCC: International Federation for Clinical Chemistry; NGSP: National Glycohemoglobin Standardization Program; DCCT: Diabetes Control and Complications Trial.

Characteristics	HemoCue® HbA1c 501 System	SD Biosensor A1c Care
Principle	Boronate affinity assay for determination of HbA1c percentage in whole blood	Immunoassay
Calibration	Factory calibrated and traceable to IFCC and NGSP/DCCT	Easy Calibration by cartridges
Sample Material	Capillary or venous whole blood	Capillary or venous whole blood
Measurement Range	20 - 130 mmol/mol (IFCC) 4.0 - 14.0% (NGSP)	20 - 140 mmol/mol (IFCC) 4.0 - 15.0% (NGSP)
Coefficient of Variation (CV)	CV < 3%	CV < 3%
Output Results	In 5 minutes	In 3 minutes
Sample Volume	4 µL	5 µl
Dimensions	198 mm (H) × 217 mm (W) × 136 mm (D)	163 mm (H) x 96 mm (W) x 56 mm (D)
Weight	1.600 kg	0.450 kg
Storage Temperature	Analyzer: 10 - 35°C (50 - 95°F) Test Cartridge: unopened 2-32°C (36 - 90°F)	0 °C - 32°C
Operating Temperature	17 - 32°C (63 - 90°F)	15 - 32°C (59 - 90°F)
Power	9 V DC / 1.5 A	12 V DC/ 1.5 A
Battery Backup	Absent	4 AA battery is used to run analyzer
Interface	Printer, PC, and Barcode Scanner	Thermal Printer, Barcode Scanner, HbA1c Management Software
Quality Control	Built-in self-test check cartridge, the system can be verified using liquid controls	Built-in self-test check cartridge
Cost of Analyzer	INR 200,000 (About 3500 USD)	INR 50,000/- (About 850 USD)
Cost per test	INR 300/- (About 5 USD)	INR 200/- (About 3.5 USD)

For all the tests we have collected the following four variables to know the feasibility of the point-of-care HbA1c assays: i) Device failure events - the device does not get powered on, or is unable to perform a measurement. ii) Test failure events - the device gives an error message or the testing cartridge is prematurely ejected and no meaningful output is received. Test failure events are expected to result in wastage of the testing strips or cartridges. We also measured the ambient room temperature of the facility where the tests were carried out, using a temperature logger device (Lascar Electronics, Erie, PA, USA).

Sample size

We have calculated sample size by using Buderer's formula for determining sensitivity [17]. Assuming the proportion of glycemic control (HbA1c ≤ 7%) to be about 40% in the participants, for a desired sensitivity of at least 90%, we needed a sample size of at least 111 participants for a relative precision of 10% of sensitivity (i.e., 95% CI for 90% point estimate to be between 81%-99%).

Statistical analysis

We have summarized nominal variables with frequency and percent and numerical variables with mean and standard deviation. Concordance in estimated HbA1c values was visualized by creating Bland-Altmann plots (mean of HbA1c by index and reference test vs difference in HbA1 between index and reference test) and scatter plots. For quantifying concordance, we have estimated Lin’s concordance correlation coefficient, Pearson’s correlation coefficient, and Area Under ROC Curve (AUC). Lin’s concordance correlation coefficient (CCC) is used to examine the agreement between two continuous measurements. Like Pearson’s correlation coefficient, CCC ranges from -1 to +1. However, if one method of measurement differs systematically from other methods then we will get a higher value of correlation coefficient, but concordance is not ensured. Therefore, Lin’s CCC is a better measurement when we aim to assess agreement or concordance. For qualitative agreement, using a cut-off of 7% we estimated traditional measures of diagnostic accuracy (Sensitivity, Specificity, Positive and Negative predictive values, and Likelihood ratios). We measured the precision of our estimates by calculating 95% confidence intervals. Data analysis was done using SPSS software (IBM SPSS Statistics for Macintosh, Version 26.0., IBM Corp., Armonk, NY) and R software.

## Results

The study was conducted between April and September 2016. A total of 114 patients (63 from urban and 51 from rural facility) were included in the study. Most patients were middle-aged (mean age 53.4 years (SD 11.5)), with an age range of 18 to 80 years. A total of 45 (39.5%) participants were women. The characteristics of the study population are detailed in Table 2.

**Table 2:** Study population (n = 114)

Variable	n	%
Female Gender	45	39.5
Complications / Comorbidities		
Retinopathy	8	7.0
Nephropathy	13	11.4
Neuropathy	23	20.2
Coronary Artery Disease	12	10.5
Cerebrovascular Disease	1	0.9
Peripheral Vascular Disease	4	3.5
Diabetic foot ulcers	2	1.8
Hypertension	23	20.2
Dyslipidemia	17	14.9
Therapies		
Metformin	69	61.1
Sulphonylurea	38	33.6
Thiazolidinedione	2	1.8
Alpha glucosidase inhibitor	3	2.7
DPP-4 inhibitors	11	9.8
Insulin	16	14.4

**Figure 1:**
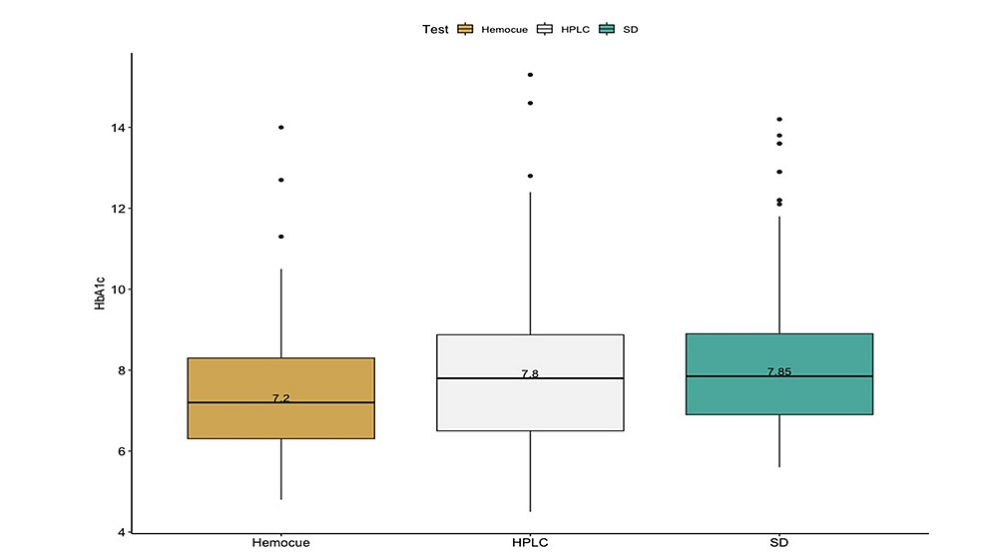
Box plot of the point-of-care HbA1c tests and reference standard. The median (inter-quartile range) HbA1c by SD device was 7.85 (6.9-8.9), Hemocue device was 7.2 (6.3-8.3) and by HPLC was 7.8 (6.5-8.9). HPLC: High-performance liquid chromatography

A valid reference standard HbA1c by HPLC method was obtained in all 114 participants. The mean HbA1c level by reference standard was 8.03% (SD 2.02). A valid HbA1c estimate by index Hemocue A1c501 and SDA1c Biosensor was obtained in 103 and 110 participants, respectively. As compared to HPLC, the median HbA1c values were similar in the SDA1c device, and Hemocue A1c device. The distribution of the HbA1c values is depicted in Figure 1.

**Figure 2:**
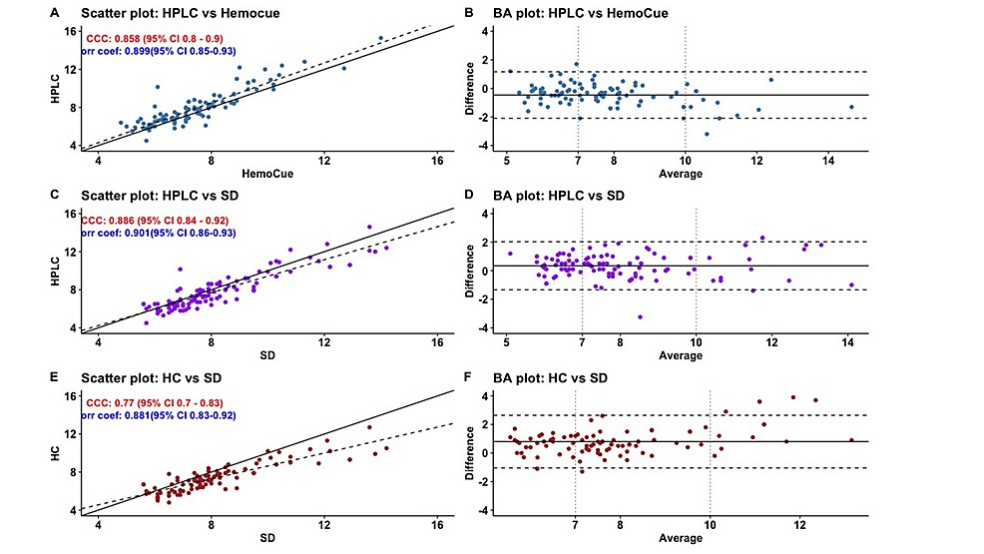
Scatter and Bland Altman plots HPLC vs. Hemocue (A-B), HPLC vs. SD (C-D) and Hemocue vs SD (E-F). Scatter plots (A, C, E) are showing measurement by one method on X-axis and by the other on Y-axis. For a good concordance, their values should be clustered near 45-degree diagonal line. Solid line indicates this 45-degree line. Dashed line is fit line for linear regression analysis. Lin’s Concordance Correlation Coefficient and Pearson’s Correlation Coefficient are also displayed along with their 95% confidence intervals. Bland Altman Plot (B, D, F) is used for visualizing the concordance between HbA1C levels estimated by two methods. The difference in HbA1C by two methods is plotted on Y-axis and the mean of HbA1C by the two methods is plotted on X-axis. If both methods are concordant then it is expected that most of the observations would line around line of 0 differences (black, bold horizontal line). In case of discordance, most values would lie beneath two standard deviations of mean differences on either side (dotted horizontal lines). HPLC: High-performance liquid chromatography; CCC: Concordance correlation coefficient.

Lin’s concordance correlation coefficient (CCC) for both SDA1c device (0.88 95% CI 0.84 - 0.92) and Hemocue device (0.85 95% CI 0.80 - 0.90) indicated good concordance. The Bland-Altmann plots (Plot of difference between index test and reference standard vs. the Mean HbA1c value by both techniques) show a majority of the mean values between the acceptable range of +/- 2SD. There is no difference in the distribution of values for lower (<7%), intermediate (7-10%), and high (>10%) HbA1c (Figure 2).

**Figure 3:**
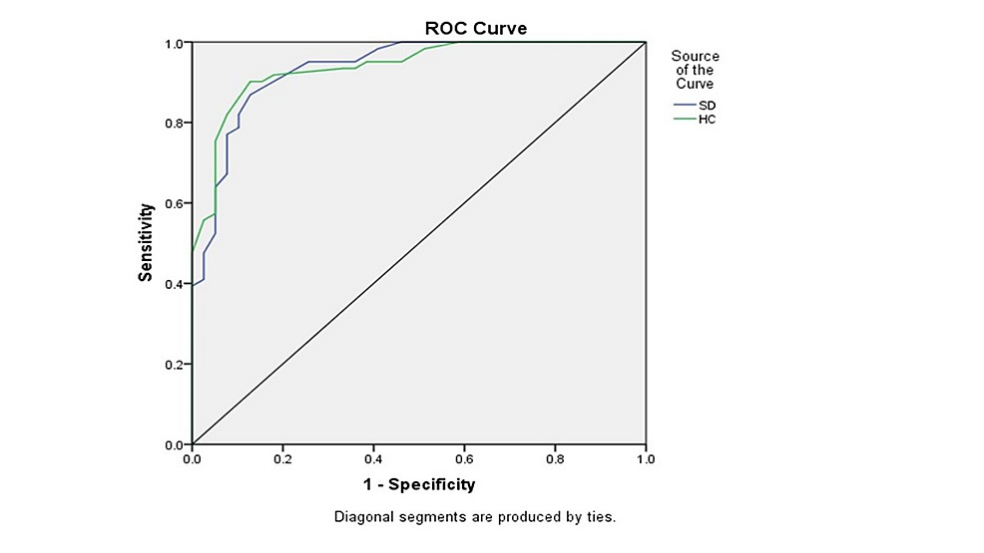
Area under the curve for SDA1c and Hemocue A1c devices for reference standard HbA1c cut-off of 7%: The Area under the curve (AUC) for SDA1c device is 0.935 (95%CI 0.886-0.983), and for Hemocue device is 0.938 (95%CI 0.893-0.984).

SD A1c assay for detection of poor glycemic control (HbA1c value >7%) was 95.38% sensitive (95% CI 87.29, 98.42), but only 68.89% specific (95% CI 54.33-80.47). In contrast, the sensitivity of Hemocue device was 90.63% (95% CI 81.02-95.63) and specificity was 87.18% (95% CI 73.29-94.2). A Hemocue device HbA1c value of above 7.0 (positive test) correctly predicted poor glycemic control 92% times (vs. 81.58% for SD device). SD device HbA1c value of less than 7.0 (negative test) correctly predicted optimal glycemic control 91% times (vs. 85% by Hemocue device) (Table 3). The overall test performance by receiver operating curve (ROC) derived analysis area under the curve (AUC) analysis was similar. The AUC for SDA1c device was 0.935 (95% CI 0.886-0.983), and for Hemocue device was 0.938 (95% CI 0.893-0.984) (Figure 3).

**Table 3:** Diagnostic accuracy of point-of-care HbA1C measurement. HbA1c of 7% or greater is considered as a positive test indicating poor glycemic control. PPV: Positive predictive value; NPV: Negative predictive value; LR+: Positive likelihood ratio; LR- : Negative likelihood ratio; HPLC: High-performance liquid chromatography.

Comparison	SD vs. HPLC (n = 110)	Hemocue vs. HPLC (n = 103)
True Positives	62	58
False Positives	14	5
False Negatives	3	6
True Negatives	31	34
Diagnostic Accuracy	84.55 (76.64, 90.12)	89.32 (81.88, 93.93)
Sensitivity	95.38 (87.29, 98.42)	90.63 (81.02, 95.63)
Specificity	68.89 (54.33, 80.47)	87.18 (73.29, 94.4)
PPV	81.58 (71.42, 88.7)	92.06 (82.73, 96.56)
NPV	91.18 (77.04, 96.95)	85 (70.93-92.94)
LR+	3.066 (2.6-3.5)	7.069 (4.76-10.5)
LR-	0.067 (0.034-0.13)	0.107 (0.076-0.15)

The device failure rate of the index tests was on four occasions with the SD A1c device, and on 11 occasions with Hemocue A1c device. We encountered 14 test failures with SD A1c device and 12 test failures with Hemocue A1c device. We could perform the test after using a new test strip in all these 26 situations. The test failures for the SDA1c device occurred at median ambient room temperature of 35°C (Range 28-35°C), and with Hemocue A1c device at a median temperature of 36°C (Range 35-37°C). However, the test non-failure events (or test success events) also occurred at the same temperature [median 37°C (Range 25-42°C) for SD A1c] and median 36°C (Range 25-42°C) for Hemocue A1c device. The mean number of test strips used to perform a single test was 1.20 with SD device and 1.19 with Hemocue A1c device.

## Discussion

In the current study, we demonstrated that both techniques for point-of-care estimation of HbA1c levels were comparable to the reference standard. The current study is one of the largest and only such work from the developing world. We encountered more device failures with the Hemocue device and comparable test failures with both devices. This provides us with important insights into the performance of point-of-care tests in actual field conditions.

Our diagnostic study included those participants who would have received the tests in actual clinical practice. Most participants in our study were middle-aged men with uncontrolled T1DM or T2DM. These demographics are comparable to previous studies in terms of age but had a lower representation of women [16]. The mean HbA1c level by reference standard in our study was 8.03% (SD 2.02), higher than in previous studies [18]. These differences are a reflection of poor glycemic control in a developing country setting. Since there was a greater variation in the HbA1c values in our study, it has a potential to bias diagnostic accuracy estimates towards the null, as compared to the studies that included participants with lower mean HbA1c values. Despite this only 2.3% and 4.8% values were outside the two-standard deviation in the Bland-Altmann plot for SD and Hemocue A1c device, respectively. This variation is comparable to previously reported studies. Previous HbA1c POC studies are listed in Table 4.

**Table 4:** Comparative analysis of previous studies of point-of-care HbA1c Immunoassays: Siemens DCA, A1cNow Borate affinity assays: BioRad Int2it HPLC: High-performance liquid chromatography

Author (Year)	Country	Participants	Index test(s)	Reference standard	Agreement
Berbudi et al., 2020 [19]	Indonesia	108	HemoCue AB	HPLC	Sn 97.83%; Sp 77.4% for HbA1c of 6.5%; Mean difference -0.187
Gomez-Peralta et al., 2016 [20]	Spain		Bio-Rad, Hercules	HPLC	Sn 85.7%; Sp 85.3%, correlation 0.712
Marley et al., 2015 [21]	Australia	241	DCA 2000+ Siemens	Cobasintegra 800	Sn 73.7%; Sp 98.2% for HbA1c of 6.4%; Correlation coefficient 0.88; Mean difference -0.15% (-0.67 to 0.36%)
Villar-del-campo et al., 2014 [22]	Spain	102	DCA 2000+ Siemens	HPLC	Correlation coefficient 0.97; Mean difference 0.024%
Knaebel et al., 2013 [23]	US	40	Bayer Aic now	HPLC	Correlation coefficient 0.98, mean difference -0.55 to +0.55%
Petersen et al., 2010 [24]	US	88	Siemens DCA, Affinion, BioRad In2it	HPLC BioRadVar 2	Mean Difference: Siemens 0.7%, Affinion 0.6%, BioRad 0%
Martin et al., 2010 [25]	France		BioRadInt 2	HPLC Variant 2	Correlation 0.97
Arrendale et al., 2008 [26]	US	70	Bayer A1c Now	HPLC	Correlation 0.89
Sicard and Taylor, 2005 [27]	US	23	Bayer A1c Now	HPLC	Correlation coefficient 0.75

These differences in the diagnostic accuracy estimates are due to small variations around the cut-off in both assay methods (SD assay with a mean 0.34 lower, and Hemocue A1c device with a mean 0.43 higher values) as compared to HPLC-based measurements. For a clinician who is assessing poor glycemic control, a Hemocue A1c measurement of 7% or higher, is 81% predictive of a similar result by HPLC in contrast to 92% predictiveness by SD assay (positive predictive value). In contrast, if the aim is to assess optimal glycemic control Hemocue A1c assay is 91% predictive in contrast to 85% by SD assay (negative predictive value). Overall both assays are similar as their AUCs in ROC analysis are similar. The overall test performances of both index tests in the current study are also comparable to a study by Marley et al. from Australia [21].

There was higher device failure with Hemocue A1c device in our study. Device failure is a condition when either the device does not switch on or is unable to receive a test strip or cartridge. The key reason for high device failure with Hemocue A1c device (11/114) was a power failure, as this equipment operates only on running electricity and does not have a battery backup. The principal reason for SD A1c device to fail was battery run-off. Availability of running electricity is a constraint, and longer battery life is an asset for any point-of-care device in a developing country setting. We encountered 14 test failures with the SD A1c device and 12 test failures with the Hemocue A1c device. Test failure is a condition where the device gives an error message after insertion of the test strip. We could perform the test after using a new test strip in all these 26 situations. The test failures were random and could not be explained by temperature and more parameters like humidity have to be taken into account in future studies. The test failure rate and test-strip/cartridge wastage were similar for both devices. While both the manufacturers suggest performing the test below a room temperature of 28°C, the ambient room temperatures were higher than this benchmark on most occasions. This is also typical of a tropical developing country scenario where POC tests are performed in a non-temperature-controlled environment. The room temperatures are above 28°C in most months of the year in our locality.

The current study was conducted in the same population that would have received the test in actual practice. The performance of the index tests and interpretation of its results was blinded and independent of the reference standard. Both the index tests and reference standard were performed using blood samples that were collected at the same time. We used clinically useful cut-offs for the interpretation of our results. The study was conducted in two different facilities, where the ambient temperature ranged from 25-42°C and the range of HbA1c tested was also wide-ranging from 4.5 to 15.3%. Our study had certain limitations. We did not study the variant hemoglobin and absolute hemoglobin level of the patients, however, this is unlikely to affect the results of our study as the prevalence of hemoglobinopathies is likely to be low. Our sample size was modest, yet this is one of the largest studies conducted. Our testing environment was at variance with what would have been prescribed by the manufacturers, but this limitation is inherent, as actual temperature and humidity levels in developing country facilities are likely to be less than ideal.

## Conclusions

Both the commercially available point-of-care HbA1c tests evaluated in this study (i.e., borate affinity or immunoassay) has similar results in comparison to the reference HPLC method. The sensitivity of the point-of-care HbA1c tests is fairly good meaning that they can serve as an acceptable alternative to the time-consuming HPLC method as a rapid screening tool. The higher positive predictive value implies we can fairly rely on the result of HbA1c > 7% for early titration of the drugs. The comparatively lower specificity and negative predictive values at HbA1c > 7% may make the clinicians watchful before changing the drugs. Both of these, point-of-care tests correlate well with the standard reference test with a wide range of temperatures (25-42°C). The temperature had no significant effect on the device and test failures.

## References

[1] Alberti KG, Zimmet PZ (1998). Definition, diagnosis and classification of diabetes mellitus and its complications. Part 1: diagnosis and classification of diabetes mellitus. Provisional report of a WHO consultation. Diabet Med.

[2] India State-Level Disease Burden Initiative Diabetes Collaborators (2018). The increasing burden of diabetes and variations among the states of India: the Global Burden of Disease Study 1990-2016. Lancet Glob Health.

[3] Wild S, Roglic G, Green A, Sicree R, King H (2004). Global prevalence of diabetes: estimates for the year 2000 and projections for 2030. Diabetes Care.

[4] Herman WH, Ye W, Griffin SJ (2015). Early detection and treatment of type 2 diabetes reduce cardiovascular morbidity and mortality: a simulation of the results of the Anglo-Danish-Dutch study of intensive treatment in people with screen-detected diabetes in primary care (ADDITION-Europe). Diabetes Care.

[5] American Diabetes Association (2013). Standards of medical care in diabetes--2013. Diabetes Care.

[6] American Diabetes Association (2015). 2. Classification and diagnosis of diabetes. Diabetes Care.

[7] Diabetes Control and Complications Trial Research Group (2017). The effect of intensive treatment of diabetes on the development and progression of long-term complications in insulin-dependent diabetes mellitus. N Engl J Med.

[8] UK Prospective Diabetes Study (UKPDS) Group (1998). Effect of intensive blood-glucose control with metformin on complications in overweight patients with type 2 diabetes (UKPDS 34). Lancet.

[9] Zemlin AE, Matsha TE, Hassan MS, Erasmus RT (2011). HbA1c of 6.5% to diagnose diabetes mellitus -- does it work for us? -- the Bellville South Africa study. PLoS One.

[10] Karnchanasorn R, Huang J, Ou HY, Feng W, Chuang LM, Chiu KC, Samoa R (2016). Comparison of the current diagnostic criterion of HbA1c with fasting and 2-hour plasma glucose concentration. J Diabetes Res.

[11] Little RR, Roberts WL (2009). A review of variant hemoglobins interfering with hemoglobin A1c measurement. J Diabetes Sci Technol.

[12] Weykamp C, John WG, Mosca A (2009). A review of the challenge in measuring hemoglobin A1c. J Diabetes Sci Technol.

[13] Karami A, Baradaran A (2014). Comparative evaluation of three different methods for HbA1c measurement with high-performance liquid chromatography in diabetic patients. Adv Biomed Res.

[14] Klenk DC, Hermanson GT, Krohn Krohn (1982). Determination of glycosylated hemoglobin by affinity chromatography: comparison with colorimetric and ion-exchange methods, and effects of common interferences. Clin Chem.

[15] Little RR (2003). Glycated hemoglobin standardization--National Glycohemoglobin Standardization Program (NGSP) perspective. Clin Chem Lab Med.

[16] St John A, Price CP (2014). Existing and emerging technologies for point-of-care testing. Clin Biochem Rev.

[17] Buderer NM (1996). Statistical methodology: I. Incorporating the prevalence of disease into the sample size calculation for sensitivity and specificity. Acad Emerg Med.

[18] Marley JV, Oh MS, Hadgraft NT, Singleton SL, Isaacs K, Atkinson DN (2015). Using glycated haemoglobin testing to simplify diabetes screening in remote Aboriginal Australian health care settings. Med J Aust.

[19] Berbudi A, Rahmadika N, Tjahjadi AI, Ruslami R (2020). Performance of point-of-care testing compared with the standard laboratory diagnostic test in the measurement of HbA1c in Indonesian diabetic and nondiabetic subjects. J Diabetes Res.

[20] Gomez-Peralta F, Abreu C, Andreu-Urioste L (2016). Point-of-care capillary HbA1c measurement in the emergency department: a useful tool to detect unrecognized and uncontrolled diabetes. Int J Emerg Med.

[21] Marley JV, Oh MS, Hadgraft N, Singleton S, Isaacs K, Atkinson D (2015). Cross-sectional comparison of point-of-care with laboratory HbA₁c in detecting diabetes in real-world remote Aboriginal settings. BMJ Open.

[22] Villar-del-Campo MC, Rodríguez-Caravaca G, Gil-Yonte P, Cidoncha-Calderón E, García-Cruces Méndez J, Donnay-Pérez S (2014). Diagnostic agreement between two glycosylated a1b hemoglobin methods in Primary Care (Article in Spanish). Semergen.

[23] Knaebel J, Irvin BR, Xie CZ (2013). Accuracy and clinical utility of a point-of-care HbA1c testing device. Postgrad Med.

[24] Petersen JR, Omoruyi FO, Mohammad AA, Shea TJ, Okorodudu AO, Ju H (2010). Hemoglobin A1c: assessment of three POC analyzers relative to a central laboratory method. Clin Chim Acta.

[25] Martin M, Leroy N, Sulmont V, Gillery P (2010). Evaluation of the In2it analyzer for HbA1c determination. Diabetes Metab.

[26] Arrendale JR, Cherian SE, Zineh I, Chirico MJ, Taylor JR (2008). Assessment of glycated hemoglobin using A1CNowTM point-of-care device as compared to central laboratory testing. J Diabetes Sci Technol.

[27] Sicard DA, Taylor JR (2005). Comparison of point-of-care HbA1c test versus standardized laboratory testing. Ann Pharmacother.

